# An *in vivo* assessment of implant stability coated with platelet-rich fibrin (PRF) using a resonance frequency analyser

**DOI:** 10.6026/9732063002001776

**Published:** 2024-12-31

**Authors:** Mahantesh Achanur, Meghna Chauhan, Kumari Lucy Bhola, Tanu Priya Sonkar, Kiran Kumar H.S, Latha K. Vel, Abhaya Chandra Das, Vardarajula Venkat Ramaiah

**Affiliations:** 1 Department of Prosthodntics and Crown and Bridge, PMNM Dental College and Hospital, Bagalkot-587101, Karnataka, India; 2 Department of Dentistry (Prosthodontics and Crown & Bridge), World College of Medical Sciences & Research and Hospital, Jhajjar, Haryana, India; 3 Department of Periodontology, Kalinga Institute of Dental Sciences (KIDS), KIIT Deemed to be University, Bhubaneswar, Odisha, India; 4 Department of Dentistry (Prosthodontics and Crown & Bridge), Autonomous State Medical College, Firozabad, India; 5 Department of Prosthodontics and Implantology, Sri Hasanamba Dental College and Hospital, Hassan, Karnataka , India; 6 Department of Periodontics, Nandha Dental College and Hospital, Tamilnadu, India; 7 Department of Periodontics and Oral Implantology, Institute of Dental Sciences, Siksha 'O' Anusandhan (Deemed to be University), Khandagiri Square, Bhubaneswar - 751030 Odisha, India; 8 Department of Dental Hygiene, College of Applied Health Sciences, ArRass Qassim University, Saudi Arabia

**Keywords:** Dental implant, platelet-rich fibrin, resonance frequency analysis, stability

## Abstract

Permanent teeth that are lost are frequently replaced by dental implants. The success of an implant is influenced by its stability. Therefore, it is of interest to assess the primary and final stability of PRF-coated implants using a resonance frequency analyser. The study comprised twenty-four healthy patients of both sexes with at least one missing tooth. They were arbitrarily allocated to two groups, each consisting of 12 samples: Group I (Control) was not given platelet rich fibrin (PRF) and Group II (study group) was given PRF. Blood was drawn from the subjects to create the platelet-rich fibrin. Resonance frequency analysis (RFA) was used to record the primary and secondary implant stability quotient values for a total of 24 implants, 12 of which were coated with platelet-rich fibrin (PRF) (test group) and 12 of which were not (control group). A statistical analysis was performed on the collected data. There were 20 patients who were male and only 4 out of 3 instances were female. Overall primary implant stability was found to be statistically not significant when comparing with and without PRF (p = 0.1268). Conversely, a statistically considerable variation (p = 0.001) was found when comparing overall secondary implant stability with and without PRF. With the PRF group, there was less marginal bone loss. This suggests that PRF increases the stability of implants. Compared to the group without PRF, implants containing PRF demonstrated better Osseo integration and less marginal bone loss.

## Background:

Implants are extensively used as a therapeutic option for the replacement of lost or missing teeth. Endosseous dental implants replace lost teeth without requiring support from neighbouring teeth, much like a natural tooth root [1]. Osteo integration is necessary for success of dental implant. The American Academy of Implant Dentistry (AAID) defines Osseo integration as the process of direct implant-bone contact for load distribution [2]. Both primary and secondary stability are involved in implant stability, which is a sign of osseo-integration [3]. Bone density attained during initial implantation and primary stability, which is impacted by implant variables. Secondary stability is influenced by surface properties and is dependent on bone remodelling [4]. Introduced in 1996, resonance frequency analysis (RFA) is a non-invasive and dependable technique for measuring stability with an implant stability quotient (ISQ), where a value of >65 indicates success and <50 indicates possible failure [3]. It assesses bone density and implant stability over time using structural and vibration principle analysis [2, 5]. This method measures implant stability by either reading an Implant Stability Quotient (ISQ) value that is supplied by the RFA (Osstell ISQ, Sweden) or by measuring the resonance frequency of the implant-bone complex [6]. Histologic examination and other clinical procedures are commonly used to assess implant stability, which is a sign of osseointegration [2]. Reverse torque testing, clinical assessment of cutting resistance during implant insertion, perio-test, the percussion test and dental fine tester are some of the techniques used to evaluate implant stability [5, 7]. While the ISQ value ranges digitally from 0 to 100, clinical values generally fall between 40 and 80 [8]. A number of techniques are used to improve the degree of bone-to-implant contact (BIC), which promotes healing and improve secondary stability. These techniques include changing the surface chemistry of the implants and introducing growth factors and biomolecules to the implant surface (such as PRF material or allograft graft). Applying cell adhesion molecules or bone morphogenetic proteins to the implant surface may improve functional integration and encourage osteoblast growth. Platelet-rich products have improved bone regeneration and accelerated implant osseointegration [5]. A polymerised fibrin matrix containing platelets, cytokines, leukocytes and circulating stem cells that release growth factors like insulin-like growth factor, transforming growth factor-β, vascular endothelial growth factor and platelet-derived growth factor-all of which aid in the healing of soft and hard tissues-makes up platelet-rich fibrin (PRF), an autologous second-generation platelet concentrate [9]. PRF serves as a resorbable membrane for directed bone regeneration and a biodegradable scaffold for wound healing [5]. In oral surgery, platelet-rich products such as platelet-rich growth factor (PRGF), platelet-rich fibrin (PRF) and platelet-rich plasma (PRP) have recently been suggested as a means of improving osseous, epithelial and tissue regeneration [2, 10]. A second-generation platelet concentrate is called as platelet rich fibrin. The PRF preparation is a straightforward procedure that involves centrifuging of human blood. The initial stage of peri-implant bone healing is the creation of a fibrin scaffold. Following this, activated platelets locally release a variety of growth factors, including platelet-derived growth factor (PDGF) and bone morphogenetic proteins (BMP). According to Öncü *et al.* PRF application seemed to improve osseointegration and bone-implant contact (BIC) during the early healing phase, hence increases implant stability [10]. Therefore, it is of interest to assess the stability of implant with PRF using resonance frequency analysis.

## Materials and Methods:

This prospective clinical research was done in Prosthodontics, crown and bridge department after obtaining ethical clearances from the concerned authority and informed consent from all the participants. The participants were included after considering the inclusion and exclusion criteria. Inclusion criteria was healed edentulous alveolar ridge requiring at least one dental implant, acceptable width and height of alveolar bone, absence of systemic conditions and medication and presence of opposing teeth. Total 24 patients of both genders were divided randomly into 2 groups with 12 samples in each as; Group I (Control) - without platelet rich fibrin (PRF) and Group II (study group) with PRF. Before stage I surgery for implant placement, a comprehensive blood investigation was done. The platelet-rich fibrin was prepared from the blood collected from the participant's venous blood. After aseptic measures, muco periosteal flap was raised and placement of PRF material in implant site was done fallowed by implant (NORIS®) placement in study group and but in control group only implant (NORIS® implant, Noris Medical Ltd., Nesher, Israel) was placed without PRF. In the second stage of implant procedure cover screw was placed and implant stability was checked. Primary implant and secondary stability for both implants (study and control implants) was measured at baseline and after 1, 3, 6 months using resonance frequency analysis (RFA) Osste llTM ISQ in a bucco-lingual and in mesio-distal directions. Implant stability quotient values were recorded for a total of 24 implants, 12 of which were coated with platelet-rich fibrin (test group) and 12 of which were not (control group). The obtained data was statistically evaluated using SPSS statistical software version 22.0 using the way paired t test with p<0.05.

## Results:

The patients were 28.3 years old on average. Only 4 three cases were female, while the 20 patients were male. On comparing overall primary implant stability with and without PRF, revealed statistically not significant (p = 0.1268). In contrast, comparing overall secondary implant stability with and without PRF revealed a statistically considerable variation (p = 0.001). The discrepancy between these figures indicates that the study group had greater secondary stability whereas the control group has more primary stability (Table 1). Marginal bone loss was lower with PRF group (Table 2). This indicates that PRF improves implant stability.

## Discussion:

Dental implants are a more recent method of replacing lost teeth. For an implant to be successful in the long run, stability is essential. On a histological and microscopical level, osseointegration is the direct interaction of living bone tissue with a dental implant without any intervening connective tissue [11]. PRF is one of various techniques that can be used to improve implant stability. Leukocytes, cytokines and stem cells combine to generate a fibrin mesh with PRF, a second-generation autologous platelet concentrate that is essential for angiogenesis and promotes both soft and hard tissue healing [12]. The form of the bone-implant contact, the amount and quality of peri-implant bone has a significant impact on the bone-implant relationship [11]. The stability of implants following PRF installation was examined in the current study both at the time of initial implant placement and six months after implantation. The results indicated that, there was higher stability using PRF compared to without PRF in implant placement. There was no discernible relationship between bone density and primary and secondary stability in both the study or control groups and the majority of the implants (24 implant fixtures) in this investigation were positioned in D3 bone type. Synergy and Meredith predicted a slight decline in stability levels if the initial ISQ is high and thus characterised ISQ ≥ 70 as strong stability [13]. According to Kapoor *et al.* PRF significantly affects dental implant osseointegration during the early healing phase before loading [11]. According to a systematic review by Pawar *et al.* the use of PRF improves implant durability and osseointegration [14]. According to Raj *et al.* applying injectable PRF to the implant site greatly improves stability six to twelve weeks after surgery [15]. According to Elsheikh *et al.*'s research, PRF can be employed as a gap filler when combined with rapid implant insertion; however, alternative bone grafting materials produce better results in terms of changes in buccal bone thickness and loss [16]. PRF may improve implant stability following implant surgery, according to Guan *et al.*'s systematic review and meta-analysis [17]. According to Qu *et al.*'s systematic review and meta-analysis, platelet concentrates can short-term decrease marginal bone loss and greatly increase implant stability [18]. These studies are consistent with what we found. Singhal *et al.* came to the conclusion that PBMSCs and platelet-rich fibrin matrix improved implant stability since they observed higher and statistically significant ISQ values after one week (p = 0.18), one month (p ≤ 0.001) and three months [19]. Anapu *et al.* used a resonant frequency analyser to assess the initial and final stability of PRF-coated implants. They came to the conclusion that implants coated with platelet-rich fibrin had superior osseointegration compared to those that were not [5]. This is in agreement with our results. Tamilarasan *et al.* evaluated the primary and secondary stability of endosseous dental implants with and without the use of Platelet-Rich Fibrin. They concluded that both groups showed considerable enhancement in the GI, PI and SBI. The PRF group showed superior IS in the 3rd third and 6thmonths [2]. The impact of local application PRF on dental implant stability was assessed by Hussien *et al.* They came to the conclusion that there was no statistically significant positive impact on implant stability from local PRF application [1]. Three months following implant installation, L-PRF did not enhance the RFA results of implants, according to Darestani *et al.* [20]. This is in contrast with the present study result. According to Bischof *et al.*'s research; bone type might not have an impact on the main stability of implants or their stability following a 12-week follow-up [21]. Peri-implantal deficiencies in T-PRF regeneration have been found by Kuzu *et al.* to be as effective as autogenous bone grafts [8]. In comparison to mandibular implants, Saracoglu *et al.* found that irradiated maxillary implants displayed statistically lower values [7]. The limitation of the current is; smaller sample size, shorter duration of follow-up time. Further studies are needed on larger samples on longer duration.

## Conclusion:

Implants with platelet-rich fibrin showed superior osseointegration compared to without PRF.

## Figures and Tables

**Table 1:** Mean (SD) of ISQ values for implant stability at baseline to 6 months

**Group**	**Primary stability**	**Secondary stability**			**p**
	**Baseline**	**1^st^month**	**3 month**	**6 month**	
Group I - Control	61.02±7.34	62.21±8.35	65.35±9.65	69±8.57	0.05
Group II - with PRF	60.24±7.65	66.53±8.44	70.45±9.76	74±8.75	
p	0.1268	0.001	0.001	0.001	

**Table 2:** Comparison of marginal bone loss among groups

**Group**	**Mean±SD**	**t**	**df**	**P**
Group I - Control	-0.358±1.35	5.364	12	0.001
Group II - with PRF	-0.217±1.32	7.356	12	
